# Anti-inflammatory and Immune Therapy for Alzheimer's Disease: Current Status and Future Directions

**DOI:** 10.2174/157015907782793667

**Published:** 2007-12

**Authors:** Douglas Walker, Lih-Fen Lue

**Affiliations:** Laboratory of Neuroinflammation, Sun Health Research Institute, Sun City, Arizona, USA

**Keywords:** Neurodegeneration, NSAIDS, cytokines, microglia, vaccination, neuroprotection, dementia, amyloid beta.

## Abstract

From the initial characterizations of inflammatory responses in Alzheimer’s disease (AD) affected brains, namely the demonstration of activated microglia and reactive astrocytes, complement system activation, increased production of proinflammatory cytokines, and evidence for microglial-produced neurotoxins, there was hope that reducing inflammation might be a feasible treatment for this memory-robbing disease. This hope was supported by a number of epidemiology studies demonstrating that patients who took non-steroidal anti-inflammatory drugs had significantly lower risk of developing AD. However, clinical trials of anti-inflammatories have not shown effectiveness, and in recent years, the concept of immune therapy has become a treatment option as animal studies and clinical trials with Aβ vaccines have demonstrated enhanced amyloid removal through stimulation of microglial phagocytosis.

This review will examine the current status of whether inhibiting inflammation is a valid therapeutic target for treating AD; what lessons have come from the clinical trials; what new pathways and classes of agents are being considered; and how this field of research can progress towards new therapeutics. We will examine a number of agents that have shown effectiveness in reducing inflammation amongst other demonstrated mechanisms of action. The major focus of much AD drug discovery has been in identifying agents that have anti-amyloid properties; however, a number of these agents were first identified for their anti-inflammatory properties. As drug development and clinical testing is a costly and lengthy endeavor, sound justification of new therapeutic targets is required. Possible future directions for AD anti-inflammatory or immune clearance therapy will be discussed based on recent experimental data.

## INTRODUCTION

Alzheimer’s disease (AD) represents one of the most serious health issues for the elderly. With the aging of the population, particularly in western societies, the increase in AD is going to be a major health-care crisis. For example, in the United States AD currently affects approximately 4.5 million, but this is predicted to rise to 16 million by 2050. Although heart disease and cancer cause higher rates of mortality, effective treatments are available for these conditions, while this is not the situation for AD. Patients diagnosed with AD are currently treated with acetylcholinesterase inhibitors (donepezil, rivastigmine or galantamine), with memantine, an NMDA receptor antagonist, being an additional treatment option for more severely-affected AD patients. These agents have been demonstrated to have a significant effect on slowing the progression of the disease, as measured by different psychometric parameters; however, it is widely accepted that their effectiveness is limited and the need for new therapeutic agents is urgent [35,58,144,184].

### Pathology of AD

AD can only be definitively diagnosed by a pathological examination of the brain postmortem. Histological examination of vulnerable brain areas of an AD case (for example, hippocampus, entorhinal, temporal, frontal and parietal cortex) with appropriate histochemical stains or immunological reagents will identify profuse numbers of extracellular amyloid beta peptide (Aβ) plaques, and also large numbers of neurofibrillary tangles (NFTs). Aβ is derived from the pathological processing of the amyloid precursor protein (APP) at the β-secretase and γ-secretase sites to produce the 4 kD Aβ fragments of 40 or 42 amino acids [64,168]. NFTs are insoluble structures and represent the remains of the cytoskeleton of dead or dying neurons. The insolubility of these structures arise on account of the hyperphosphorylation of the microtubule-associated protein tau [47]. With the availability about 20 years ago of appropriate antibodies that could localize proteins in formaldehyde-fixed brain tissues, studies identified the presence of a type of chronic microglial inflammatory response in AD brain tissues, especially associated with the hallmark plaque and tangle pathological structures [83,104,116]. Activated microglia were particularly evident using antibodies that recognize the class II major histocompatibility complex protein HLA-DR. The insolubility and persistence of these pathological structures appears to incite this microglial inflammatory response, though cellular contents of damaged cells also contribute to microglial activation; microglia being a population of brain-resident cells with most of the characteristics of macrophages/monocytes [178]. The normal function of microglia within the brain is immune surveillance; they are the first cell type to respond to any perturbation or injury within the CNS. Although T lymphocytes, B lymphocytes and peripheral macrophages have been identified in AD brains [49,82,147], it appears that the inflammatory response in the brain neuropil is not primarily being mediated by these invading peripheral immune cells, but by brain-resident cells. A major discriminating feature of AD inflammation compared to multiple sclerosis is the lack of significant numbers of infiltrating leukocytes. As the inflammation in AD is not being driven by IFN-γ  [149], it appears to develop and persist in a chronic manner. Increased expression by microglia in AD brains of the cytokine IL-1 was also initially reported as a feature of microglial activation [66]. Other features of an immune response in AD brains include activation of the complement system, which has the potential to further damage neurons and amplify any ongoing inflammatory response. Plaques and tangles in AD brains are immunoreactive for activation fragments of the complement pathway [41, 114,145]. From these pathological observations, a wide range of experimental studies using cultured microglia isolated from rodent or human brains demonstrated that Aβ peptide, when in an aggregated, fibril or oligomeric form, could activate microglia to a proinflammatory state (reviewed in [178]), which included increased production of proinflammatory cytokines, reactive oxygen species, degradative proteases and neurotoxic factors [16,54,62,177]. The characterization of inflammatory responses in postmortem brain led to an “inflammatory hypothesis” for AD, which suggested that the inflammatory factors produced in the brain, particularly as a result of microglial activation by Aβ, could be causing a loss of healthy neurons or damaging axonal processes or synapses. Positron emission tomography studies of living AD patients with the ligand PK11195, which binds to the peripheral benzodiazepine receptor and specifically identifies activated microglia, has shown greater binding in neocortical areas of brains of AD subjects, even patients with early stage AD [21,175]. This is indicative of inflammation being an event occurring early in the pathogenesis of the disease [21,175].

### Risk Factors for AD

AD is the most common form of dementia, accounting for up to 60% of all cases. Aging is the most important risk for developing AD, as its incidence is around 1% in the 60-64 year age-group, but rising to about 30% in those aged 85 years or older. Associated risk factors are head trauma, small brain size, female gender, low educational status and reduced physical and mental ability in later life [20,57,98]. The major genetic risk factor is possession of the apolipoprotein E (apoE) ε4 allele [110]. Other risk factors of AD, all of which are associated with vascular disease, and some that can be affected by possession of the apoE ε4, include diabetes, hypercholesterolemia, hypertension, coronary heart disease, obesity, smoking and atherosclerosis [36]. A strong association between cerebral atherosclerosis and the development of AD pathology has recently been demonstrated [13,23,148]. To date, the only consistent data from case-control, cross-sectional epidemiological, and prospective incidence studies of subjects concerning what reduces the risk of AD were a history of taking NSAIDS (examples [17,18,22,80, 97,117]). Although some of these earlier studies have limitations due to possible selection biases [97], the Baltimore Longitudinal Study of Aging, a prospective incidence study, did confirm a reduced risk of AD in subjects taking NSAIDS [163]. Similarly, the Rotterdam Study of Aging prospective study has demonstrated a relative risk of 0.2 (with range 0.05-0.83 at 95% confidence interval) for developing AD in those taking NSAIDS for more than 24 months [81].

There is no doubt that the pathogenesis of AD is multifactorial involving a combination of genetic factors and non-genetic components, which together can lead to the generation of toxic processes involving dysregulation of amyloid precursor protein metabolism, inflammation, oxidative stress, apoptosis, proteosome inhibition, mitochondrial dysfunction, imbalances in antioxidant production, and glutamate excitotoxicity, amongst others [133]. As such, it appears important that new therapeutic agents have multiple targets of action that affect a number of these processes.

### Inflammation and AD

A comprehensive summary of inflammation and AD was compiled about 7 years ago, with the conclusion that inflammation must be contributing to AD pathology on account of the many toxic inflammatory proteins that are upregulated in AD brains [123]. With the apparent lack of effect in clinical trials of tested agents (primarily COX inhibitors) on slowing the progression of mental deterioration, we should consider whether inflammation is still a relevant target for AD. A scheme for how inflammation and Aβ could interact to cause AD pathology is presented in Fig (**1**). This model makes the assumption that the initiating events for neuropathology in the brain start in the periphery (i.e. vascular inflammation, atherosclerosis, coronary heart disease) and lead to the initial Aβ deposition; this progression of events is not universally accepted [201]. Aβ deposition due to reduced clearance can initiate a cascade of events leading to accelerated Aβ oligomerization and aggregation that can directly cause synaptic loss and neurodegeneration, and ultimately dementia. Aβ is considered by most as the primary driving force of AD, due to its neurotoxic, oxidative stress and proinflammatory effects on multiple cell types. There is evidence that cerebral inflammation can lead to increased Aβ production [77]; the question that remains to be answered is to what extent inflammatory factors produced in brain directly lead to synaptic damage and neurodegeneration; answering this question is central to deciding which anti-inflammatory agents should be tested.

To be effective, the appropriate anti-inflammatory agent must be administered to the appropriate clinical population, who are not too far advanced in the disease. If one considers at what stage of the disease to administer anti-inflammatory therapeutics, Fig. (**2**) is derived from data from two publications on the relative staging of microglia, plaques and NFT in hippocampus and entorhinal cortical sections of subjects with progressively increasing clinical dementia rating (CDR) scores [170,187]. There is a progressive increase in each of these parameters as dementia increases, though both studies show a decline in microglia scores as the neuritic plaque progresses from stage 3 to 5, or NFT progresses from 5 to terminal stage 6. This would indicate that microglia activation could “burn out” at the last stages of pathology once mental decline can not be further measured. It would appear that at early and moderate stages of AD, there is the potential to reduce inflammation, and plaque and tangle formation, using drugs that target all features of this pathology.

### Clinical Trials of Anti-Inflammatory Agents with AD Subjects

Prospective double-blind placebo-controlled trials are considered the standard for determining whether an agent is effective for a particular disease. A number of trials of anti-inflammatory agents have been carried out with AD subjects. The design, dosage, degree of severity and inclusion criteria of the patient population was not consistent between the trials. The first such trial for AD of a NSAID involved a small group of patients treated for 6 months with indomethacin or placebo [146]. The indomethacin-treated patients showed no decline in mental function, while the control group declined by 8.4 %. A pilot trial of the NSAID diclofenac in combination with misoprostol, to provide gastrointestinal protection, in a group of mild to moderate AD patients showed no significant difference in indices of cognitive decline between treated and placebo subjects after 25 weeks [155]. A small scale study of the NSAID nimesulide, a preferential COX-2 inhibitor, for 24 weeks showed no significant differences in rates of cognitive changes [6]. A larger scale trial for 1 year of refocoxib, another selective COX2 inhibitor, or of naproxen, a non-selective COX inhibitor, versus placebo was ineffective at slowing cognitive decline [5]. The lack of effect of refecoxib was confirmed in a 1-year trial of mild to moderate AD subjects [139]; similarly the anti-inflammatory agent hydroxychloroquine showed no protective effect in preventing decline in memory function in a large 18-month trial [171]. A trial of low-dose prednisone, a potent steroid anti-inflammatory, for 1 year showed no difference in cognitive decline between the treated and placebo groups [4].

The pronounced dichotomy that has still to be resolved is that epidemiological data showing that subjects taking NSAIDs for more than 2 years are protected from AD [81,97,117,195], while the clinical treatment trials of anti-inflammatories with diagnosed AD subjects have generally been negative in preventing cognitive decline. It had been hoped that the Alzheimer Disease Anti-Inflammatory Prevention Trial on normal elderly with a family history of AD would determine whether NSAIDs could prevent or delay the onset of AD [118]. However, these will remain unanswered as this trial with celecoxib, naproxen, or placebo was terminated before completion due to cardiovascular and cerebrovascular side-effects from the active drugs, particularly naproxen [1].

## THERAPEUTIC AGENTS AND TARGETS FOR ALZHEIMER’S DISEASE

At the experimental level, new classes of therapeutic agents are being examined that have one or several targets in the pathways believed to be involved in AD pathogenesis. Agents being tested at different levels include those that inhibit the β-  and γ- secretase enzymes, thus preventing Aβ peptide being formed [76,96]; statin agents that lower circulating levels of cholesterol, but which also have anti-inflammatory properties [38]; agents that inhibit the aggregation of Aβ into toxic oligomers and fibrils [61]; agents that inhibit kinases (e.g. glycogen synthase kinase (GSK) 3 and cell division cycle kinase (CDC) 25) that are involved in the phosphorylation of tau [15]; cytoskeletal modifying agents; thiazolidinedione agents used to treat type 2 diabetes; and anti-oxidants and anti-inflammatory agents [200]. A number of agents being tested for effectiveness in AD, for example statins and curcumin have activities against multiple relevant targets in pathways involved in AD pathogenesis. Fig. (**3**) outlines the properties and pathways that an “ideal” AD treatment agent might need to modulate in order to modify AD pathogenesis. Data from studies with these different types of agents will be discussed to consider why future clinical trials of agents with anti-inflammatory properties should focus only on those with multiple additional relevant mechanisms of action.

### Inhibition of Cyclooxygenase

The majority of the anti-inflammatory agents tested on AD patients are prostaglandin H synthase (COX) inhibitors. Although some of these agents have other less-well defined modes of actions, as trials with these agents have not shown positive results, it might indicate that inhibition of the prostaglandin pathways might not be a suitable target for retarding the pathogenesis of established AD [72]. The role of COX metabolites in AD pathogenesis is still unclear. In human brain, COX-2 is primarily localized to neurons, and not in activated microglia as would be expected [71,73]. Induction of COX-2 immunoreactivity in neurons of AD brains is an early event in the disease pathogenesis being maximal at Braak tangle stage 0-II before microglial activation is a prominent feature [74], but declining as the disease progresses [192]. Measurements of COX-2 mRNA in AD brains generally showed increased levels associated with pathology [108,130,191]. As some COX metabolites (prostaglandins PGD_1_ PGD_2_, PGJ_2_, and 15d-PGJ_2_) are agonists for PPAR-γ, activation of which can induce anti-inflammatory pathways in macrophages, microglia and astrocytes, inhibition of synthesis of proinflammatory PG (e.g. PGE_2_) will also reduce the amounts of anti-inflammatory PG. Similarly, PGE_2_ has a role in neuroprotection; treatment of primary neurons with PGE_2_ or agonists for the EP-2 and EP-4 PG receptors resulted in significant neuroprotection from Aβ toxicity [40], an effect mediated by increased intracellular levels of cyclic AMP. Activation of microglia EP2 receptor with PGE_2_ increased Aβ phagocytosis and reduced the microglial-mediated neurotoxicity [159]. Crossing transgenic mice overexpressing COX-2 in neurons with a line of Aβ plaque-developing mice (APPswe/PS1-A246E) resulted in enhanced production and deposition of Aβ(40) and Aβ(42) at 24 months [189], but not at 12 months [188], although a greater percentage of hippocampal neurons from COX2APPswe/PS1-A246E mice showed apoptosis in response to Aβ(1-42) than neurons from APPswe/PS1-A246E mice. Thus, it can be seen that the role of COX and inhibition of PG synthesis in AD pathology is complex; inhibiting the production of PGs with protective/anti-inflammatory properties might be more detrimental for chronic AD pathogenic events than preventing the effects of proinflammatory PGs. Even though the trials with agents that had preferential COX-1 inhibiting activity were generally unsuccessful, a role for COX-1 in AD inflammation is still possible as COX-1 immunoreactivity is found in microglia in AD brains, particularly in microglia associated with amyloid plaques [73,193].

### Non-Steroidal Anti-Inflammatory Drugs (NSAIDS)

Most NSAIDS - aspirin, ibuprofen, indomethacin, sulindac, and flurbiprofen - have inhibition of COX as their major mechanism of anti-inflammatory activity. These agents, most of which are available without prescription, have preferential activity in inhibiting COX-1, but also have some activity against COX-2, a form of the enzyme primarily induced during inflammatory responses. The new generations of COX-2 specific inhibitors, which appear effective for controlling arthritis, have also been considered as AD therapeutic agents. In recent years, Aβ lowering or aggregation inhibition mechanisms of action of NSAIDs have been identified that are not related to COX inhibition or anti-inflammatory properties. In a series of studies, treatment of cultured cells (neural and non-neural) with diclofenac, flurbiprofen (R and S and mixed enantiomers), ibuprofen, indomethacin, sulindac sulfide and meclofenamic acid resulted in lower levels of Aβ(42), but not Aβ(40), production [43,181] by modulating γ- secretase activity [14,34]. In order of effectiveness at lowering Aβ 1-42 production by H4 cells were flurbiprofen, meclofenamic acid, sulindac, fenoprofen, indomethacin, diclofenac and ibuprofen [43]. Agents that showed no activity in this assay included acetaminophen, aspirin, dapsone, fenbufen, ketoprofen, meloxicam and sulindac or sulindac sulfone [43]. The effectiveness of flurbiprofen, ibuprofen and sulindac sulfide for lowering Aβ production has been confirmed using primary neurons as the target cell [56], but this study did not report the specific effect to Aβ(42) as these agents were effective at lowering Aβ(40) production as well. These authors concluded that the mechanism of action of these NSAIDs is not related to their COX, lipoxygenase, NFκB or IκB inhibiting-, or PPARγ-activating-, properties, as specific agents for these targets did not affect Aβ levels in the assays used [151]. The effect appeared to be due to inhibition of Rho and a Rho-kinase [199]. Aβ-lowering activity was also being observed using specific Rho-kinase inhibitors, although cells deficient in Rho-kinase activity did not demonstrate reduced amounts of Aβ production. The NSAIDs that demonstrated significant Aβ-lowering properties in cultured cells were also effective in lowering Aβ (1-42) in brains of transgenic Tg2576 mice [43]. By contrast, another study that administered indomethacin or the COX-2 inhibitor nimesulide to Tg2576 mice for 8 months, from 7-15 months of age, showed significant Aβ-lowering effect with indomethacin alone [165]. These authors suggested that the effect was due to inhibition of NFκB as cells deficient in NFκB activity did not show the same response. Certain NSAIDs also exert anti-inflammatory activity through activation of the nuclear hormone receptor class of transcription factors PPAR [99]. PPAR-γbelongs to this group of nuclear receptors that include PPAR-α and PPAR- δ , which control lipid and glucose metabolism, energy levels, monocyte differentiation and inflammatory responses. As mentioned in a previous section, natural ligands for PPAR-γinclude certain COX and lipoxygenase metabolites, but of the NSAIDs, indomethacin has highest affinity and selectivity for PPAR-γ, with diclofenac, ibuprofen, flufenamic acid having lower affinities. PPAR can function through several mechanisms, including formation of heterodimers with retinoid-x-receptors [166]; these complexes bind to PPRE and activate transcription of certain genes. Inhibition of inflammatory processes is believed to occur by a mechanism of receptor-dependent transrepression, whereby activated PPAR-γinteracts with other transcription factors or transcriptional co-activators, to prevent the activation of inflammatory-associated transcription factors such as NFκB, STAT-1, C/EBP and AP-1 from activating inflammatory gene expression [60].

### Thiazolidinediones

Thiazolidinediones, which are prescribed for the treatment of type 2 diabetes to increase insulin sensitivity, are PPAR agonists. Agents under investigation as potential AD therapeutics include pioglitazone, rosiglitazone, troglitazone and ciglitazone, which also act as agonists for PPAR-α, but with lower degrees of affinity. However, they can also induce significant anti-inflammatory activity on microglia and macrophages through this pathway. PPAR-γagonists ibuprofen, indomethacin, pioglitazone, ciglitazone, and 15d-PGJ_2 _were effective in preventing toxicity to cultured neurons from media of Aβ-stimulated monocytes or microglia [31]. Treatment of murine microglia and astrocytes with 15d-PGJ_2_, rosiglitazone, pioglitazone, or ciglitazone reduced secretion of nitric oxide, TNF-α, IL-1β, IL-6 and MCP-1, with the natural ligand 15d-PGJ_2 _being more effective than the thiazolidinediones [164]. Two studies using a rodent model of focal cerebral ischemia demonstrated that pioglitazone [109] and rosiglitazone [198] have significant acute neuroprotective activities. Treatment of lesioned animals downregulated microglial activation, reduced levels of inflammatory cytokine synthesis, and reduced infarct volumes in both studies. Treatment of 10 month-old APPV717I transgenic mice with ibuprofen or pioglitazone for only 7 days reduced numbers of activated microglia and astrocytes in cortex and hippocampus. These mice also showed significant reduction in COX-2 and iNOS mRNA, BACE-1 mRNA and protein levels, and reduction in area occupied and staining intensity of Aβ(42) plaques. Pioglitazone-treated animals showed a 27% reduction in levels of soluble Aβ (42) [70]. Anti-inflammatory properties for PPAR-γligands on expression of myeloperoxidase (MPO) mRNA were shown in granulocyte-macrophage colony stimulating factor (GM-CSF) treated human macrophages and macrophages from MPO expressing mice, though these same agents had a stimulatory effect on MPO expression when applied to macrophage colony stimulating factor (M-CSF) treated macrophages [95]. A role in AD for MPO, an enzyme secreted by phagocytic cells that catalyzes the production of the potent pro-oxidant hypochlorous acid from hydrogen peroxide, has been suggested as it colocalizes with plaques and plaque-associated microglia in AD brains, and can be expressed by Aβ- stimulated microglia *in vitro* [140]. Aberrant induction of MPO in AD brains has the potential to contribute to oxidative stress. Two studies have shown that polymorphisms in the promoter gene of MPO that alter its levels of expression were more abundant in female AD patients [140], or those showing cognitive decline [135].

A clinical trial with rosiglitazone was carried out for 24 weeks on mild to moderate AD patients. Overall there was no significant improvement in treated patients using the ADAS-Cog test, though it was shown that there was a significantly different response to the drug depending on whether the subject possessed an apoE ε4 allele [142]. ApoE ε4 positive patients continued to show cognitive decline, while apoE ε4 negative patients showed slight improvement.

### Polyphenolic Anti-Oxidants

#### Curcumin

**Fig. (3):**
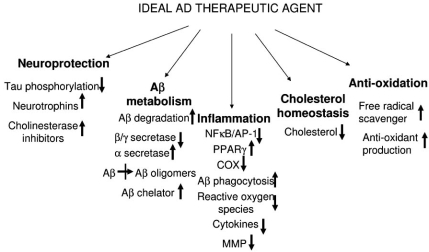
Potential targets that an *“ideal AD therapeutic agent”* might modulate to reduce the progression of AD pathology.

There has been much attention on the yellow curry spice curcumin as a therapeutic agent for AD [9,11,30,101]. When one considers the properties of a hypothetical ideal agent for treating AD (Fig. **3**), curcumin has many features that meet these requirements. Curcumin, an extract of turmeric, is a non-flavonoid polyphenol and has been widely used as a safe food additive for many centuries, particularly in India. It has been reported that AD incidence is significantly lower in Asian-Indian populations [25], who use this spice extensively in food. The biochemical pathways affected by this agent are extensive, and many of these could be of significance in inhibiting AD pathological changes. Curcumin has identified anti-inflammatory properties due to inhibiting activation of the NFκB, AP-1 and STAT inflammatory pathways [30,88,90, 91,161]. It also has defined anti-oxidant properties [51,111,137,154,185, 190], which can be partially due to the induction of anti-oxidant defensive genes heme oxygenase-1, glutathione S transferase and quinine reductase in oxidatively-stressed neurons [154], and heat shock proteins [111]. Other properties relevant to AD include cholesterol-lowering activity [134], iron chelation [86], Aβ aggregation inhibiting properties [127,190] and inhibition of expression of MMP-1, -3, -9, -14 [91]. *In vitro*, curcumin inhibited the formation of Aβ fibrils and oligomers from Aβ(40) and Aβ(42), and also induced dissociation of preformed fibrils [127,190]. Curcumin reversed markers of oxidative stress in brains of mice caused by treatment with the dopaminergic neurotoxin 1-methyl-4-phenyl-1,2,3,6-tetrahydropyridine (MPTP) [137]; reversal of oxidative stress due to traumatic brain injury in rats has been reported [185]. A study involving feeding curcumin to transgenic APP (Tg2576) mice in their diet for 6 months demonstrated significant reduction in Aβ load, and in levels of the proinflammatory cytokine IL-1β and numbers of activated microglia and reactive astrocytes [101]. A more recent study demonstrated that curcumin fed orally to aged Tg2576 mice for 5 months reduced Aβ levels and plaque load [190]. It was also shown in this study that curcumin could be localized to cerebral plaques. Administration of curcumin to aged rats injected intracerebrally with Aβ reduced oxidative damage, memory deficits and synaptophysin loss  [30,52]. Clinical trials sponsored by the National Institutes of Health on the effectiveness of curcumin as a treatment for AD are underway [2,141]. It is not clear whether the anti-amyloid properties of curcumin are more significant than the anti-inflammatory or anti-oxidant properties, but having a combination of all should be beneficial in inhibiting different pathological processes.

#### Resveratrol

Resveratrol is the principal non-flavonoid polyphenol found in grapes and red wine and, similar to curcumin, possesses a range of pharmacological properties including anti-oxidation, anti-inflammation, neuroprotection and inhibition of Aβ aggregation [7,112, 143,153,158]. Both compounds have similar chemical structures. Resveratrol has anti-inflammatory properties due to its activation of SIRT-1, a class III histone deacetylase; activation of SIRT-1 by resveratrol inhibited NF-κB signaling by promoting deacetylation of a lysine residue on RelA/p65 [194]. Its effectiveness in inhibiting Aβ-stimulated microglia-mediated neurotoxicity through this mechanism has been demonstrated [27]. Neuronal protection from Aβ toxicity, along with promotion of clearance of Aβ peptides, are additional properties associated with resveratrol [84,112]. Interestingly, although moderate wine consumption has been associated with some protection from AD [138], due to the low amounts of resveratrol in red wine, other components have also been implicated. Administration of red wine to transgenic Tg2576 mice was effective in lowering plaque load, even though the amount of resveratrol present in the wine was considered to be too low to be therapeutically effective [180].

### Statins

Statins are widely used to lower circulating levels of cholesterol through their activity as HMG-CoA reductase inhibitors and are the primary treatment for reducing the risk of coronary heart disease. High levels of circulating cholesterol are considered a risk factor for developing AD due to its effect on accelerating atherosclerosis and vascular inflammation, both of which can promote production of Aβ. There has been much interest in the use of statins as preventive therapy for AD, although the epidemiological data are conflicting to their effectiveness at lowering the risk of dementia [87,100, 196,197]; however, as a recent clinical trial of mild AD patients with atorvastatin demonstrated promising improvement in certain cognitive parameters, further trials of statins are underway [162]. A number of studies have also shown that high cellular cholesterol levels promote the β secretase pathway of Aβ formation and reduce the α-secretase formation of soluble APP (examples [93,129]). Treatment of hippocampal and cortical neurons with simvastatin and lovastatin reduced amounts of secreted Aβ(40) and Aβ(42), while treatment of guinea pigs with simvastatin reduced cerebral and CSF levels of Aβ(40) and Aβ(42) [45].

There are now well characterized anti-inflammatory properties for statins that are distinct from their cholesterol-lowering properties. These effects include lowering circulating levels of C-reactive protein [24], reducing Aβ-stimulated expression of IL-1β and iNOS in cultured macrophages or microglia [32], and reducing expression of IL-6 by rodent microglia [102].

Lovastatin, simvastatin, pravastatin and atorvastatin were all shown to have significant activity of lowering expression of myeloperoxidase (MPO) mRNA expression by human and murine macrophages, adding evidence for the anti-inflammatory properties of these agents [95]. In addition, simvastatin lowered MPO mRNA and enzyme activity even after 1 day in human MPO overexpressing transgenic mice fed drug for 1- 21 days [95]. 

Lovastatin was effective in reducing the severity of EAE through reduction in the number of infiltrating T cells and monocytes and reduced secretion of inflammatory cytokines [122]; one mechanism appeared to be the reduction of endothelial cell adhesion molecules through inhibition by lovastatin of the phosphoinositide 3 kinase-Akt (protein kinase B)-NFκB pathways.

Differences were also seen in statin effectiveness in AD mice models. One study showed that lovastatin and pravastatin lowered Aβ levels in TgCRND8 mice [26], while lovastatin increased Aβ levels in the brains of female Tg2576 mice, but not male animals [128]. Treatment of non-transgenic mice with atorvastatin, simvastatin, or lovastatin lowered endogenous levels of Aβ 40 and 42 [19].

### Antibiotics

#### Minocycline

Minocycline is a tetracycline family antibiotic widely prescribed for treating acne skin condition, but also for respiratory and neurological infections. However, its other identified anti-inflammatory and neuroprotective properties have indicated its possible use for treating neurodegenerative diseases including AD. Minocycline was effective in reducing inflammatory cytokines IL-1β, IL-6, and TNF- α levels in an AD transgenic mouse model, and improving cognitive performance though not reducing Aβ levels [157]. Also, minocycline reduced IL-6 and TNF-α production by Aβ-stimulated human microglia [44]; this study also demonstrated that minocycline *in vitro* could inhibit Aβ aggregation. Minocycline has shown activity as a neuroprotective agent through inhibition of microglial activation in a number of animal disease models of neurodegeneration or neuronal damage. Minocycline slowed disease progression in a transgenic model of amyotrophic lateral sclerosis [94]. In a model of spinal cord injury, animals administered minocycline showed reduced neuronal apoptosis, reduced amounts of caspase-3, reduced microgliosis and increased functional recovery [48]; one suggested mechanism of action was reduced TNF-α production by microglia. In three different PD animal models of dopaminergic cell loss, induced by thrombin [28], paraquat [136], or MPTP [186], administration of minocycline inhibited microglia activation, and reduced free radical production by inhibiting the microglial NADPH oxidase respiratory, iNOS and cytokine production, which resulted in significant reduction in the loss of dopaminergic neurons.

#### Dapsone

Interest in the anti-leprosy antibiotic dapsone (4,4'-diamino-diphenylsulfone) as an AD therapy came from initial observations of lower incidence of AD in a population of Japanese leprosy patients, who had been treated for many years with this or similar anti-leprosy antibiotics [115]. Pathological examination of brains of leprosy patients indicated significantly fewer amyloid plaques than in age-matched controls [79,121], though the numbers of NFTs were either unchanged or increased; however, other studies did not confirm these findings [65,92]. Dapsone was not effective in lowering production of Aβ(42) in an *in vitro* assay [43]. An additional mechanism of action for dapsone has been proposed, besides its antibiotic properties, namely as an anti-inflammatory. Dapsone has significant anti-inflammatory properties as an inhibitor of the enzyme myeloperoxidase (MPO) [89,173], but a small clinical trial of dapsone in AD patients was unsuccessful [79].

### Neuro-Receptor Modulators

#### Nicotine

Smoking is a significant risk factor for the development of AD [3,105], likely due to the effects of the many toxic components of cigarette smoke (e.g. carbon monoxide, phenols, formaldehyde, benzene etc) on the vasculature, and can promote atherosclerosis, oxidative stress and hypertension, all risk factors for AD. However, nicotine alone has many potential therapeutic properties. Nicotine is a potent ligand for a large family of NAChR that are expressed in brain and the peripheral nervous system. Tg 2576 transgenic mice administered acute and chronic doses of nicotine showed significantly less accumulation or load of Aβ in their brains [67,125]; this effect was not replicated in a different triple transgenic (APP/tau) mouse model [126], where non-significant changes in Aβ levels were detected in treated animals, and where nicotine increased the proportion of phosphorylated tau. However, recently a significant reduction of Aβ(40) and Aβ(42), both soluble and insoluble forms, was demonstrated in APP(V717)L transgenic mice administered nicotine for 5 months from 9 to 14 months of age. Accompanying these findings was the demonstration of significant reduction of levels of activated NFκB and MAPK pathway components. Inhibition of these pathways resulted in downregulation of expression of apoptosis and cell cycle genes, and reduced amounts of the inflammatory-associated iNOS [103].

It has been shown that smokers with AD had significantly less soluble and insoluble Aβ(40) and Aβ(42) in hippocampus and temporal cortex brain samples than non-smokers with AD [68], while smoking non-demented controls had significantly less soluble Aβ(40) and Aβ(42) than non-smoking controls. Although these data do not indicate a protective role for smoking in AD, they do suggest nicotine as a therapeutic agent for AD warrants further investigation. This agent can not only inhibit Aβ aggregation and prevent its resulting cytotoxicity *in vitro* [119], but it has significant demonstrated anti-inflammatory properties. Nicotine in combination with galantamine (an acetylcholinesterase inhibitor that has nicotinic receptor binding activity) inhibited microglia activation induced by HIV gp120 and IFN-γ[63]; in another study, nicotine or acetylcholine significantly inhibited microglia secretion of tumor necrosis factor-α (TNF-α) induced by LPS [160], an effect mediated by inhibiting the phosphorylation of p42/44 ERK1/2 and p38 MAPK, and attenuated by the NAChR α-7 antagonist alpha bungarotoxin. Rodent microglia were demonstrated to express NAChR α-7 mRNA by RT-PCR [160]. Treatment of rodent microglia with nicotine was effective at reducing LPS-induced TNF-α secretion, but also induced expression of COX-2 and production of PGE_2_. NAChR α-7 is the receptor mediating the anti-inflammatory effects of acetylcholine or nicotine on macrophages, as NAChR α-7 deficient mice did not show reduced TNF-α secretion in response to acetylcholine stimulation [179]. Human monocytes responded to nicotine by downregulation of proinflammatory cytokines mediated by inhibition of IκB phosphorylation and reduced transcription of NFκB. A role for acetylcholine in mediating the “cholinergic anti-inflammatory pathway” has been established in the periphery, for example bacterial peritonitis in mice was exacerbated if cholinergic vagus input was prevented [172], and reduced following nicotine administration. An early deficit in acetylcholine has been repeatedly demonstrated in AD [50], and is the target for currently prescribed acetylcholinesterase drugs to enhance acetylcholine neurotransmission. These recent data indicate that an acetylcholine deficit may contribute to perpetuating the chronic inflammation in AD brains. These anti-inflammatory mechanisms could function in human brains as we show in Fig. (**4**) that NAChRα-7 mRNA is expressed by human microglia derived from postmortem elderly brain, while NAChR α-4 and NAChRβ2 are not.

## IMMUNE THERAPY AND AMYLOID SEQUESTRATION

The other side of the issue of anti-inflammatory therapy for treating AD is the use of immune therapy to induce circulating antibodies to the Aβ peptide so that they can either bind and sequester the circulating Aβ from the blood [37], inhibit Aβ fibrillogenesis or toxic oligomer formation [53], or bind to plaques in the brain and stimulate Fc-γreceptor mediated phagocytosis by microglia [12]. It appears that all three mechanisms could be involved in immune clearance of Aβ in mice [120]. The initial findings were that immunizing PDAPP plaque developing mice with aggregated Aβ(42) to raise an antibody response to the peptide prevented Aβ deposition if mice were immunized at a young age, or aided in the clearance of Aβ deposits if mice were immunized at an older age [156]. These findings opened up the concepts of immune stimulation as a therapeutic approach to AD in a manner that would previously have been considered as pathogenic. These findings were widely replicated in different transgenic mouse models of AD (reviewed in [59]) with either active peptide immunization or passive transfer of antibodies, many of which demonstrated improvement in memory tasks in immunized mice. Utilizing microglia to phagocytose antibody-opsonized Aβ through binding to their Fc-γimmunoglobulin receptors does involve cellular activation with transient increased production of free radicals and proinflammatory cytokines [10,106]. As microglial activation appears necessary for efficient clearance of plaques, this strategy has the potential to exacerbate ongoing neuroinflammatory processes before the benefits of Aβ removal are realized.

Due to the dramatic reduction of Aβ observed in vaccinated mice, human clinical trials of the Aβ vaccine (AN1792) on human subjects were carried out up to the phase IIa stage, when they were halted due to meningoencephalitis developing in 18 of 298 vaccinated subjects. It appeared that a T-cell mediated autoimmune response was responsible for the inflammatory response. Although the vaccine trial was terminated, the brains of certain vaccinated participants became available for pathological studies. These demonstrated extensive regional plaque clearance [113,124], with evidence for microglial phagocytosis of Aβ; however, tangles were not cleared from the cortex and there was persistence of cerebral amyloid angiopathy. A recent study of Aβ species in the brains of two additional AN1792 vaccinated subjects demonstrated that although plaques had been dissolved, the total load of Aβ was not reduced, but moved into a soluble oligomeric form. These cases also had high levels of Aβ in the white matter, and pronounced deposition of Aβ on vessels. These data indicate that antibody-mediated mobilization of Aβ from plaques has the potential to transform Aβ into more toxic and inflammatory soluble oligomeric forms [131].

## AD ANTI-INFLAMMATORY DRUG DISCOVERY

Identifying new relevant targets using cell culture models for a particular disease provides the basis for drug discovery of potentially specific anti-inflammatory targets for AD inflammation. Our laboratory took the approach of global gene expression profiling employing human postmortem brain-derived microglia stimulated with aggregated/oligomeric Aβ (1-42) to identify all possible genes induced by this interaction [177]. The use of human postmortem microglia is a well-established model of microglial interactions with Aβ plaques for studying the activation of microglia by aggregated Aβ [106,107,140,176]. Our laboratory has shown that blocking the Aβ-binding receptor for advanced glycation endproducts (RAGE) on Aβ- treated human microglia has significant anti-inflammatory properties [107]. RAGE, which is upregulated on a number of cell types in AD brains including microglia, astrocytes, vascular cells and neurons, is currently a drug target for AD, and a number of other vascular and inflammatory diseases [75]. We have also used an expression profiling approach, the simultaneous induction by microglia of multiple inflammatory pathways was demonstrated; these included a range of inflammatory cytokines (e.g. IL-1β, IL-6) chemokines (e.g. IL-8, MCP- 1, -2, -3, MIP -1α, -1β, -2α, -2β, -3α;), proteases (e.g. MMP -1,, -3, -9, -12), enzymes (e.g. COX-2, indoleamine-pyrole 2,3, dioxygenase) and inflammatory receptors (e.g. urokinase plasminogen activator receptor and immunoglobulin Fc-γ receptor-IIa) [177]. In this model for inflammation in the AD brain, we identified a range of genes whose expressions were induced more than 3-fold in all of the 5 separate isolates of microglia used in the study. Although the changes in gene expression by human microglia following activation by Aβ were predominantly proinflammatory, we also identified a limited number of potentially anti-inflammation molecules that were induced in parallel. These included IL-1 receptor antagonist, somatostatin receptor-2, vitamin D receptor, endothelial cell protein C receptor, and adenosine 2A receptor. Upregulation of these proteins on microglia, particularly the anti-inflammatory receptors, as a result of Aβ stimulation suggests a potential target for downregulation of the inflammation by administering the receptor ligand or agonist. All of these targets have been characterized in different inflammatory paradigms, but not as potential therapeutic targets for AD. For example, somatostatin receptor-2 is the most abundant of the somatostatin receptors expressed by inflammatory cells [42]; activation of somatostatin receptors with somatostatin or agonists can downregulate proinflammatory cytokine secretion by human macrophages, epithelial cells or rodent microglia [8,29,46,169]. In keeping with our hypothesis that an AD drug should have multiple therapeutic mechanisms, it was demonstrated that somatostatin treated cortical neurons upregulate the expression of the Aβ peptide degrading protease neprilysin. A mouse model with a genetic deficiency of somatostatin had reduced neprilysin and increased Aβ (42) [152]. As somatostatin levels decline in aging and AD brains, supplemental therapy for somatostatin is considered a feasible therapeutic option [33,39]. We also propose the vitamin D receptor as a novel AD inflammatory target. It is a member of the large family of nuclear receptor transcription factors and specifically binds the micronutrient-derived hormone 1α, 25-dihydroxyvitamin D3. Vitamin D is an essential factor in stimulating or maintaining bone formation; however, this agent has also been shown effective in inhibiting inflammation [69,150,174]. For example, 1α, 25-dihydroxyvitamin D3 treatment of IFN-γstimulated macrophage inhibited the macrophage respiratory burst and reduced expression of a number of IFN-γinduced inflammatory genes [69]. Also, vitamin D3 was effective in protecting culture neurons from the toxic effects of glutamate or reactive oxygen species [78,167]. *In vitro*, vitamin D3 treatment of rats with chronic relapsing EAE was effective in reducing disease severity by inhibiting microglia and T cells activation and iNOS expression [55]. Although use of the active agent may not be possible due to the side-effect of hypercalcemia, vitamin D agonists lacking these properties, such as elocalitol, have anti-inflammatory properties *in vivo* [132].

## CONCLUSIONS

It is still not possible to conclude if anti-inflammatory treatment alone is no longer a valid approach for treating AD. It seems that the central focus of pharmaceutical drug development will remain on inhibiting Aβ production and aggregation, with anti-inflammatory properties of any such agents being a bonus. As discussed, there is a large body of experimental data from animal or cell culture models demonstrating that Aβ-  or cytokine-activated microglia can produce factors that are neurotoxic, epidemiological data showing the protective effect from developing AD of taking NSAIDs, pathological studies of postmortem AD brains showing many features of inflammation; and that anti-inflammatory drugs are effective for treating other neurodegenerative diseases or disease models, e.g., multiple sclerosis, HIV-associated dementia, PD, ALS, or stroke. All of the above suggest (*but do not prove*) that an inflammatory component must be involved in the pathogenesis of AD. It is clear that cerebral or peripheral inflammation can be an early event in the AD degenerative process, occurring before memory loss; however, separating the inflammatory pathology from the effects of Aβ on neurotoxicity and neuroinflammation may not be possible, or necessary. Many of the NSAIDs that have been shown to be effective in protecting against AD in epidemiological studies, though not in clinical studies, also have defined Aβ-lowering properties [14,43,85,182,183]. Although, it has been suggested that the effective doses of NSAIDs so as to have Aβ-lowering properties were above the physiological doses that can be used to treat patients, the effect of long-term use of these drugs at lower doses on Aβ production in presymptomatic subjects remains to be determined. Much research is still being carried out on producing and testing a modified Aβ vaccine, or testing whether passive immunization with anti-Aβ antibodies have similar Aβ clearing effects. This approach might always have limitations as it is focused on the single target of Aβ removal and actually has a proinflammatory effect due to the stimulation of microglia to phagocytose antibody-opsonized plaques.

At present, the agent curcumin, which ironically has been available for centuries as a food additive, appears to have the great potential, based on convincing experimental data of its efficacy as an anti-oxidant, anti-inflammatory and anti-amyloid agent, to be an effective AD therapeutic agent. The phase II clinical trial of curcumin is scheduled to be completed by December 2007 [2].

## Figures and Tables

**Fig. (1):**
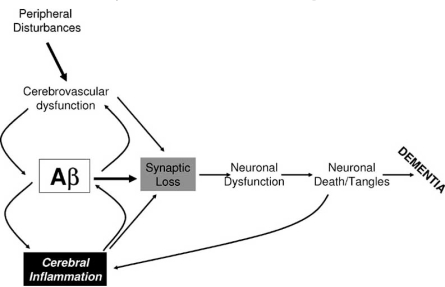
Potential interactions of Aβ peptide, vascular inflammation and cerebral inflammation in precipitating AD pathogenesis. In this scheme, peripheral disturbances (e.g. high levels of cholesterol, vascular inflammation, atherosclerosis) lead to compromise/inflammation of the cerebrovasculature. This will reduce transport of oxygen and glucose, creating conditions of oxidative stress that can increase Aβ production and aggregation, and neuronal stress directly. Deposition of Aβ can lead to cerebral inflammation, which can feedback to increase production of Aβ. The contribution of cerebral inflammation separate from Aβ to the progression of pathological changes is unclear, but inflammatory factors can mediate many of the neurotoxic events occurring in AD (tau hyperphosphorylation, synaptic loss, neuronal cell death).

**Fig. (2):**
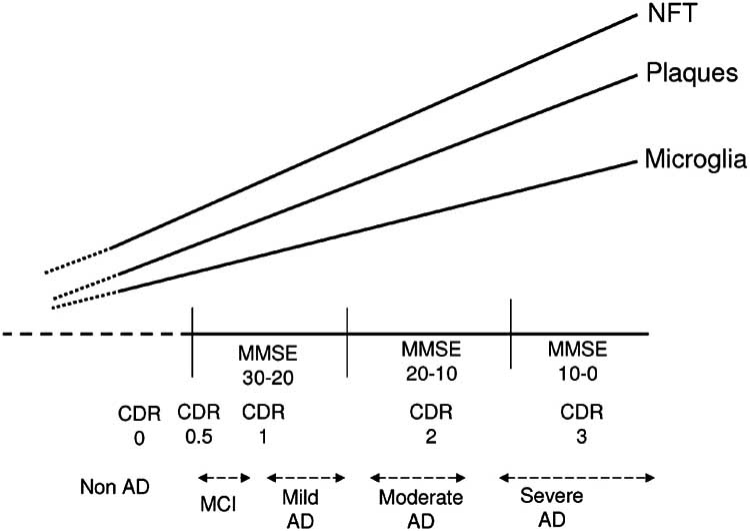
When is the optimal stage of the disease to most effectively treat AD with anti-inflammatory agents. Figure shows progression of AD pathology, namely that the density of plaques, NFTs and activated microglia increase in the hippocampus/entorhinal cortex with decline in cognitive performance. MMSE – mini-mental status exam; the primary physician screening test of patient for cognitive decline. CDR – clinical dementia rating; a more detailed patient and informant assessment of cognitive decline.

**Fig. (4):**
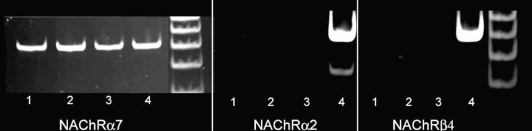
Human microglia express mRNA for nicotinic acetylcholine receptor α7. Panel A –Reverse transcription-polymerase chain reaction showing expression by different isolates of human microglia of α7 nicotinic acetylcholine receptor mRNA (NAChRα7) (lanes 1-3). Panels B and C: show that human microglia do not express α2 (NAChRα2) or β4 (NAChRβ4) nicotinic acetylcholine receptor mRNA (lanes 1-3). As positive controls (panels A, B, C: lane 4), cDNA from a sample of differentiated SH-SY5Y neuronal-like cells showing positive expression of nicotinic acetylcholine receptor α7, α2 and β4.

## ABBREVIATIONS

Aβ: = Amyloid beta
AP-1: = Activator Protein-1
APP: = Amyloid precursor protein
APOE: = Apolipoprotein E
AD: = Alzheimer’s disease
BACE-1: = Beta-Site APP-Cleaving Enzyme 1
COX: = Cyclooxygenase
EAE: = Experimental Autoimmune Encephalomyelitis
HIV: = Human Immunodeficiency Virus
IFN-γ: = Interferon gamma
IL: = Interleukin
iNOS: = Inducible Nitric Oxide Synthase
LPS: = Lipopolysaccharide
MAPK: = Mitogen-Activated Protein Kinase
MCP: = Monocycte Chemoattractant Protein
MMP: = Matrix Metalloproteinase
MIP: = Macrophage Inflammatory Protein
NAChR: = Neuronal Acetylcholine Receptor
NFTs: = Neurofibrillary tangles
NFκB: = Nuclear Factor κB
NSAIDS: = Non-steroidal Anti-inflammatory Drugs
PD: = Parkinson’s disease
PG: = Prostaglandin
PPAR: = Peroxisome proliferator-activated receptor
STAT: = Signal Transducer and Activators of Transcription
TNF: = Tumor Necrosis Factor
